# The relationships between neuroinflammation, beta-amyloid and tau deposition in Alzheimer’s disease: a longitudinal PET study

**DOI:** 10.1186/s12974-020-01820-6

**Published:** 2020-05-06

**Authors:** Rola Ismail, Peter Parbo, Lasse Stensvig Madsen, Allan K. Hansen, Kim V. Hansen, Jeppe L. Schaldemose, Pernille L. Kjeldsen, Morten G. Stokholm, Hanne Gottrup, Simon F. Eskildsen, David J. Brooks

**Affiliations:** 1 Department of Clinical Medicine, PET-Centre, Aarhus University, Aarhus, Denmark; 2 Department of Nuclear Medicine and PET Centre, Aarhus University Hospital, DK-8200 Aarhus N, Denmark; 3 Dept. of Neurology, Aarhus University Hospital, Aarhus, Denmark; 4 Centre of Functionally Integrative Neuroscience (CFIN), Aarhus University, Aarhus, Denmark; 5 Institute of Neuroscience, University of Newcastle upon Tyne, Tyne, UK; 6 Department of Medicine, Imperial College London, London, UK

**Keywords:** Alzheimer, Neuroinflammation, β-amyloid, Tau, Microglia, PET, MCI, PK11195, PiB, Flortaucipir

## Abstract

**Background:**

The aim of this longitudinal study was to assess with positron emission tomography (PET) the relationship between levels of inflammation and the loads of aggregated β-amyloid and tau at baseline and again after 2 years in prodromal Alzheimer's disease.

**Methods:**

Forty-three subjects with mild cognitive impairment (MCI) had serial ^11^C-PK11195 PET over 2 years to measure inflammation changes, and ^11^C-PiB PET to determine β-amyloid fibril load; 22 also had serial ^18^F-Flortaucipir PET to determine tau tangle load. Cortical surface statistical mapping was used to localise areas showing significant changes in tracer binding over time and to interrogate correlations between tracer binding of the tracers at baseline and after 2 years.

**Results:**

Those MCI subjects with high ^11^C-PiB uptake at baseline (classified as prodromal Alzheimer’s disease) had raised inflammation levels which significantly declined across cortical regions over 2 years although their β-amyloid levels continued to rise. Those MCI cases who had low/normal ^11^C-PiB uptake at baseline but their levels then rose over 2 years were classified as prodromal AD with low Thal phase 1-2 amyloid deposition at baseline. They showed levels of cortical inflammation which correlated with their rising β-amyloid load. Those MCI cases with baseline low ^11^C-PiB uptake that remained stable were classified as non-AD, and they showed no correlated inflammation levels. Finally, MCI cases which showed both high ^11^C-PiB and ^18^F-Flortaucipir uptake at baseline (MCI due to AD) showed a further rise in their tau tangle load over 2 years with a correlated rise in levels of inflammation.

**Conclusions:**

Our baseline and 2-year imaging findings are compatible with a biphasic trajectory of inflammation in Alzheimer’s disease: MCI cases with low baseline but subsequently rising β-amyloid load show correlated levels of microglial activation which then later decline when the β-amyloid load approaches AD levels. Later, as tau tangles form in β-amyloid positive MCI cases with prodromal AD, the rising tau load is associated with higher levels of inflammation.

## Background

Alzheimer’s disease (AD) is a neurodegenerative disorder characterized clinically by progressive impairment of cognitive functions and, in particular, early involvement of short-term memory. The pathological hallmarks of AD are the presence of extracellular beta-amyloid (Aβ) fibrillar plaques and intraneuronal neurofibrillary tau tangles (NFT). Brain slices of AD cases show that both extracellular Aβ plaques and neurons containing NFTs are surrounded by activated microglia, the intrinsic cellular immune inflammatory response to brain injury [1, 2]. The role and the trajectory of this inflammation are still being debated [3]. Activated microglia can exhibit a protective or neurotoxic phenotype depending on their environment. The protective phenotype is phagic, clearing Aβ fibrils and neuronal debris, remodelling synapses and releasing growth factors. In contrast, the neurotoxic phenotype releases cytokines such as TNFα and IL1β which can cause or contribute to tissue damage and disease pathology so driving disease progression [4].

It is thought that Aβ aggregation begins in a preclinical phase of Alzheimer’s disease and rises over a decade, approaching a plateau in the late prodromal phase when mild cognitive impairment (MCI) is present [5, 6]. Hyperphosphorylation of tau and its aggregation to form NFTs begins during the prodromal phase of AD causing neuronal dysfunction, cognitive decline and progression to clinical dementia [7].

The amyloid cascade hypothesis [8] posits tau pathology that occurs downstream from the deposition of Aβ plaques. PET studies report that cortical tau tangles are only detected when Aβ deposition is already present [9]. The link between Aβ and tau aggregation may involve activation of microglia. Soluble Aβ oligomers have been reported to activate microglial cells in a cell culture study [10]. Transgenic Alzheimer mouse studies have shown that activation of microglia precedes tau aggregation [11] and promotes tau hyperphosphorylation via cytokine release with subsequent formation of NFTs [12].

It has been suggested that brain inflammation initially occurs as a protective response against dementia, the microglia phagocytosing Aβ fibrils, but this fails, and the microglial activity declines. Later, as tau tangles accumulate, a second phase of increasing microglial activation occurs which is neurotoxic and this phenotype drives disease progression [3, 13, 14].

PET imaging biomarkers to detect Aβ-fibrils, tau-tangles and the translocator protein (TSPO) expressed by activated microglia have made it possible to examine in vivo the longitudinal time course of the inter-relationships between Aβ and P-tau aggregation and the associated neuroinflammation. Using PET to serially map the relative spatial and temporal distributions of Aβ, tau and inflammation in prodromal Alzheimer’s disease should further our understanding of the roles of inflammation in driving or protecting against Alzheimer’s disease progression.

We previously reported that at baseline 60% of our MCI subjects showed a raised brain Aβ load, and thus represented prodromal Alzheimer’s disease (pAD) cases—80 % of these pAD subjects also showed clusters of brain inflammation [15]. A correlation between cortical Aβ load and levels of microglial activation could be detected in a number of these clusters. This cross-sectional baseline study found no significant association between tau tangle load and inflammation levels in early MCI [16]. Here, we report inflammation levels 2 years later in our MCI cohort and their relationship with Aβ plaque and tau tangle loads.

Using PiB, flortaucipir and PK11195 PET imaging, our objectives were to:

1. Determine the longitudinal changes over 2 years in levels of fibrillar β-amyloid, tau tangles and inflammation (activated microglia) in mild cognitive impairment (MCI) cases.
2. Investigate the association between levels of fibrillar β-amyloid and inflammation in MCI cases who have a low/normal PiB PET signal at baseline but which subsequently rises as β-amyloid fibrils are deposited over the next 2 years. These cases are considered to have early prodromal Alzheimer’s disease (AD) with Thal phase 1-2 amyloid pathology missed by amyloid PET when applying a conventional PiB threshold for abnormality and are tau negative.
3. Examine the associations between levels of inflammation, β-amyloid load and tau tangle load in MCI cases who have a high β-amyloid signal approaching Alzheimer levels at baseline. These subjects have prodromal AD, and a majority show a rise in tau levels over 2 years.

We hypothesised that:

- Inflammation levels in MCI cases with a high β-amyloid load will decrease over 2 years as the activated microglia fail to clear amyloid and tau.
- The subgroup of MCI cases with low β-amyloid at baseline which then becomes elevated will show inflammation levels that rise alongside their increasing β-amyloid load over 2 years.
- MCI cases with a β-amyloid load approaching Alzheimer levels at baseline will show an increasing tau tangle load over 2 years. These cases will also show rising inflammation levels but these correlate with tau tangle rather than their β-amyloid load.

## Methods

### Study population

Participants were recruited from Memory/Dementia clinics in Denmark or by advertisement and were screened/assessed as previously described [15]. Our Alzheimer’s disease cases were formally diagnosed by these clinics in Denmark in accordance with the ICD-10 clinical criteria while the MCI subjects fulfilled the Petersen clinical criteria [17]—we applied no formal diagnostic rating threshold for the severity of their cognitive deficits. During the recruiting period, we ascertained a total number of 43 MCI subjects. The programme was initially designed to longitudinally investigate the inter-relationship between Aβ fibril load and levels of inflammation in MCI subjects. During the conduct of the study, ^18^F-Flortaucipir PET also became available to image tau tangles. The PET scans were scheduled to all be performed within 10 weeks, but for six subjects, the flortaucipir scan was performed up to 1 year later than the two other scans due to delayed tracer availability (see Additional file 1).

### Cognitive assessment

All subjects were cognitively rated by trained research assistants under the supervision of an experienced psychologist. Cognitive status was rated with the Montreal Cognitive Assessment (MoCA). We performed no inter- or extrapolation for missing data. To protect against bias, our cognitive assessments were performed by raters blinded to all other aspects of the subject’s condition and scan findings.

### Image processing

MRIs were acquired with a Skyra 3 Tesla system (Siemens, Erlangen, Germany), and PET scans were acquired with a high resolution research tomograph (ECAT HRRT; CTI/Siemens, Knoxwille, TN, USA)—both according to previously described protocols [15]. Target doses of 400 MBq ^11^C-PiB, ^11^C-PK11195 and 370 MBq ^18^F-Flortaucipir were intravenously administered as boluses followed by a 10-ml saline flush. PiB PET was acquired for 50 min 40–90 min post injection. Flortaucipir PET was acquired for 40 min 80–120 min post injection. The 60-min ^11^C-PK11195 emission PET scans were initiated with a 30-s ‘background’ frame prior to tracer injection. The dynamic scan was acquired in list mode, and data were re-binned as 1 × 30 s, 6 × 10 s, 2 × 30 s, 2 × 60 s, 3 × 120 s, and 10 × 300 s ‘background’ time frames.

The MINC software (http://en.wikibooks.org/wiki/MINC) [18] was used to segment MRI volumes into images of grey (GM) and white (WM) matter and cerebrospinal fluid (CSF) [19] and to spatially normalise the MRI and PET images into MNI space [20]. GM masks were convolved with a probabilistic atlas [21] to define regions of interest (ROIs) on the individual’s GM template.

The spatially normalised PiB and flortaucipir images were summed from 60–90 to 80–100 min, respectively, and voxel signals were divided by the mean signal from the individual’s cerebellar GM to generate PiB and flortaucipir standardised uptake value ratio (SUVR) images. Cases were categorised as high or low Aβ load based on the bimodal distribution of group baseline data—there was a clear separation between raised and normal PiB MCI ranges above and below a composite cortical:cerebellar PiB SUVR of 1.5 [15]. The composite cortical PiB uptake was defined as the volume weighted average of temporal, parietal and frontal GM area uptake [15].

PK11195 binding potential (BP_ND_) maps were generated at a voxel level using the simplified reference tissue model [22] implemented in MATLAB. As all anatomical regions in the brain can contain voxels with specific PK11195 binding in Alzheimer’s disease, a Supervised Cluster Analysis (SVCA) with 6 classes [23] that was applied to the dynamic images to localise clusters of voxels which either contained specific tissue binding or which provided a reference tissue input function representing normal GM uptake kinetics. Signal from voxels with the kinetics of vascular endothelial uptake was discarded. The PK11195 images were spatially normalized into MNI space in the same manner as described for the PiB images. PK11195 images were smoothed with a 6-mm FWHM Gaussian filter prior to parametric analysis, see also [15].

### Cortical surface mapping

Vector based cortical surface statistical mapping was used to localise subject and mean group significant differences in tracer uptake over time and correlations between levels of tracer binding. Cortical surfaces were generated with FACE (fast accurate cortex extraction) [24, 25]. In FACE, topologically correct surface meshes are iteratively fitted to the WM-GM and the GM-CSF interface with sub-voxel precision using pre-processed T1 weighted images [26]. FACE has been shown to be robust, accurate and fast [27].

Cortical surfaces were transformed into PET native space using the transformation matrix from a rigid body co-registration between PET and T1 weighted images [20]. PET parameters were interpolated and mapped to the surface approximating the middle cortical layer in order to minimize the influence of partial volume effects. Individual surfaces were registered to the cortical surface of an average non-linear anatomical template in MNI space [28] using a feature driven surface registration algorithm [29]. Parameter values were then mapped to the average surface and smoothed using a 20-mm FWHM geodesic Gaussian kernel. Smoothing along the cortical surface eliminates the unwanted blurring across gyri caused by smoothing in voxel space.

### Statistical analyses

STATA 14.2 (stataCorp LP, TX, USA) and Prism 8 (GraphPad Software. La Jolla, CA, USA) were used for statistical analyses. Differences in non-imaging variables between the high and low PiB groups were assessed using the general linear model, while a Wilcoxon rank-sum test was used to interrogate skewed variables. *P* values < 0.01 were considered statistically significant to correct for multiple comparisons when interrogating non-imaging parameters. The subjects’ demographics and cognitive status are summarised descriptively in Table 1.

**Table 1:** Demographic and cognitive data

	High PiB group	Low PiB group	*P* value
Baseline (*n*)	27	16	
Female, %	32.1 %	50%	0.29
Age (years)	73 ± 6.0	66 ± 8.6	*0.0025*
Education	12.5 ± 2.9	12.2 ± 3.4	0.74
Follow-up (months)^a^	24.3 ± 1.2	24.9 ± 2.6	0.32
ApoE4	64.3% (*n* = 26)	18.8% (*n* = 15)	*0.009*
MoCA			
Baseline	23.4 ± 3.3	25.7 ± 2.8 (*n* = 15)	0.025
Follow-up	21.4 ± 4.9	25.2 ± 3.4	*0.005*

^a^Between PiB scan dates. Data presented as mean ± sd. *n* number, *MoCA* montreal cognitive assessment

Cortical surface statistical maps of mean differences in tracer binding parameters were calculated at each surface vertex using a vertex specific general linear model implemented in Python. Cortical areas showing correlations between binding parameters were interrogated using the general linear model. All statistical maps were initially thresholded at *P* = 0.05 to visually localise areas of potential significant change*.* These areas then had a family wise error (FWE) rate correction applied using the cluster-extent-based thresholding software and a primary cluster-defining threshold of *p* < 0.05*.* The cluster-extent threshold was calculated using Gaussian random field implemented in SPM12 using the estimated intrinsic smoothness based on residual maps [30]. The Visbrain software was used to visualize cortical surface maps (https://www.frontiersin.org/articles/10.3389/fninf.2019.00014/full).

### Determining Aβ status

Our MCI cohort showed a bimodal distribution, with a clear separation of high and low PiB uptake clusters at a baseline composite cortical:cerebellar PiB SUVR of 1.5 [31].

## Results

### Characteristics of the studied populations

The characteristics of our study population are summarized in Table 1.

The baseline MCI cohort comprised 43 cases (mean age 70 years; range 50–83) which had both PiB and PK11195 PET and a subgroup of 25 subjects who also had flortaucipir PET. Twenty-seven (63%) of the 43 MCI cases had a composite cortical PiB SUVR > 1.5 at baseline and were categorized as high PiB representing prodromal Alzheimer’s disease. Thirteen of these 27 high PiB cases had follow-up PET with all three tracers.

The follow-up cohort comprised of thirty-eight of the MCI cases which had longitudinal PET with PiB and PK11195, and 22 of these cases had additional longitudinal PET with flortaucipir.

The high and the low PiB uptake MCI groups were similar with regard to gender, years of education and follow-up time. The baseline high PiB MCI cases showed a significantly higher prevalence of ApoE4 carriage than the low PiB MCI cases (64.3% v 18.8%; *p* = 0.009), a lower baseline MoCA score and a greater cognitive decline—see Table 1.

### Changes in binding of the three radiotracers from baseline to 2-year follow-up in the high- and low-PiB MCI groups

**Fig. 1:**
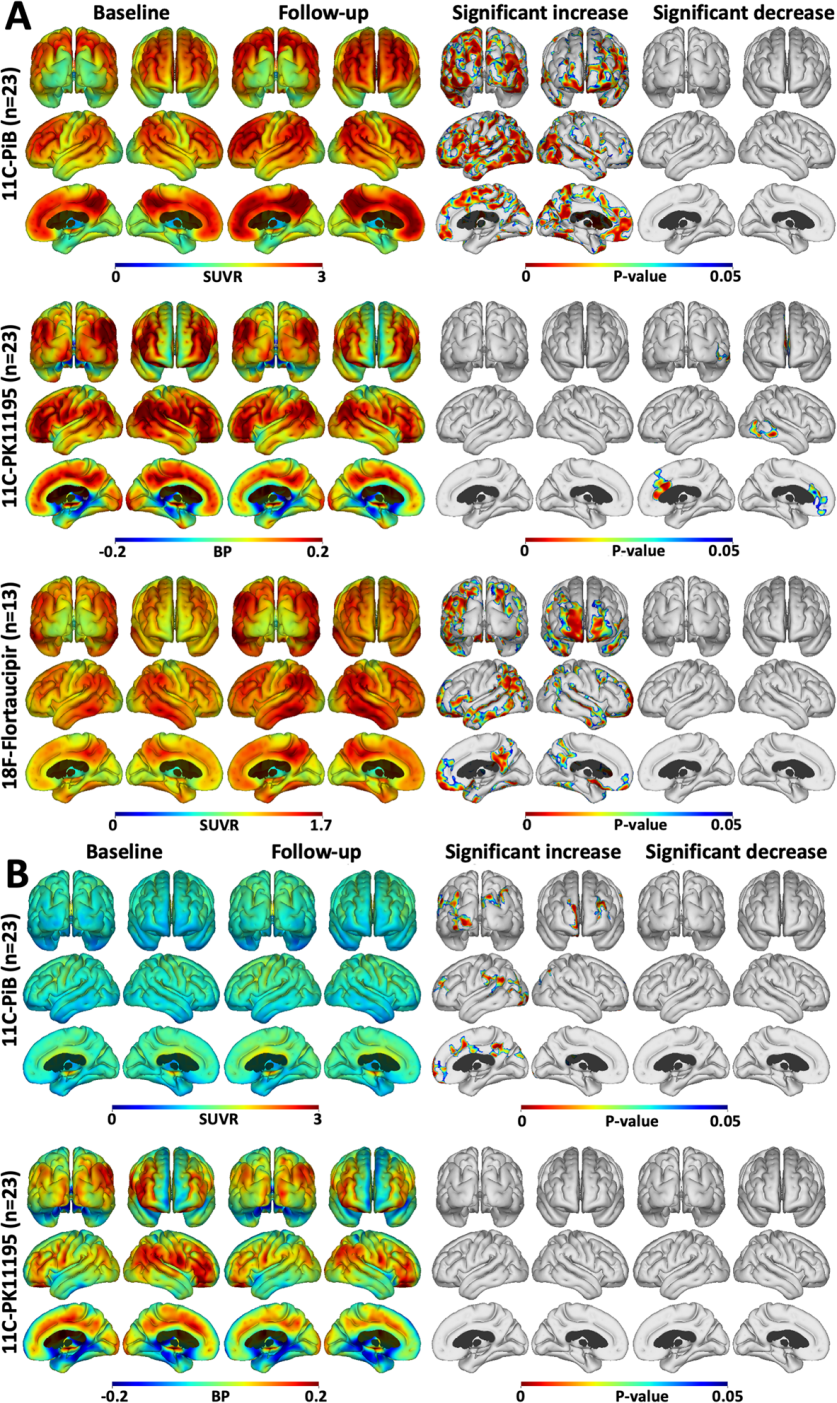
Cortical surface maps of mean tracer uptake and changes. **a** Cortical surface maps of mean PiB (upper), PK (middle) and flortaucipir (lower row) uptake for the high PiB MCI subjects at baseline and after 2 years follow-up. On the right, results of a paired *t* test between baseline and follow-up show increased amyloid in all cortical association areas, increased tau in frontal and occipital cortical areas, and reduced inflammation in fronto-temporal cortical regions after 2 years. **b** Mean PiB (upper) and PK (lower) uptake for the low PiB subjects at baseline and after 2 years follow-up. The results of a paired *t* test show small areas of increased amyloid, but no changes in inflammation levels over 2 years (*P* < 0.05; cluster FWE rate, *P* < 0.05)

All cortical surface statistical mapping results were determined using an uncorrected threshold of *P* < 0.05 to initially localise areas of potential significant change followed by an FWE rate correction of *P* < 0.05 applied to clusters to confirm the presence of significant change. The longitudinal changes in PiB, PK11195 and flortaucipir levels are presented for the baseline high PiB MCI group in Fig. 1a. This prodromal AD cohort showed areas of significantly increased cortical PiB SUVR and flortaucipir levels at a 2-year follow-up compared with baseline while the level and extent of the PK11195 signal had decreased. The cortical increase in PiB SUVR levels after 2 years was widespread, while flortaucipir increases were seen in discreet areas of frontal, temporal and parietal lobes. Cortical PK11195 binding decreases were bilateral with a fronto-temporal emphasis.

Our low baseline PiB MCI group showed no significant flortaucipir signal at baseline—see Fig. 1b in [16]. The longitudinal changes in PiB and PK11195 for the entire low PiB group of 15 MCI cases are presented in Fig. 1b. Small areas of significant increase in PiB uptake were seen reflecting the contribution of seven subjects who individually showed a rise in their cortical PiB uptake over the 2-year interval, three reaching the 1.5 SUVR threshold. We have previously reported this significant difference in baseline levels of brain inflammation between the high and the low PiB MCI cohorts [15].

### Correlations of PiB and PK11195 in the low-PiB MCI group

**Fig. 2:**
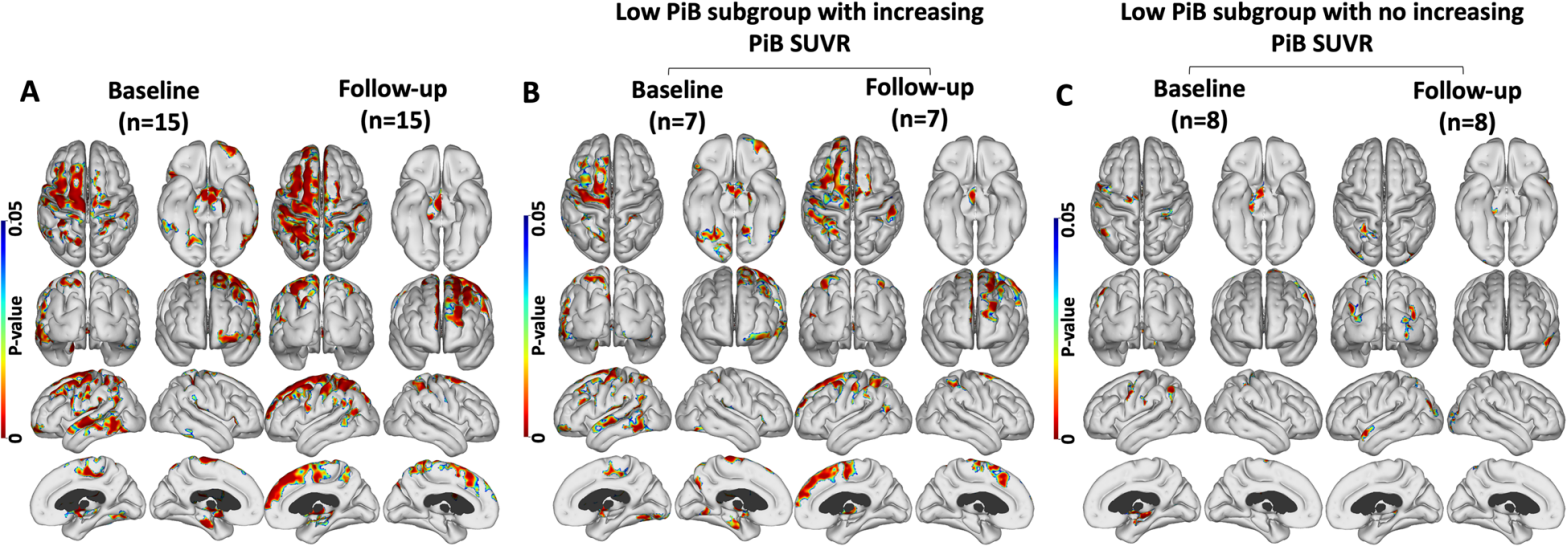
Amyloid and inflammation correlations in the low PiB group. Regions where uptake of PiB and PK are positively correlated in the low PiB group at baseline and after 2 years. **a** Across the 15 low PiB MCI subjects. **b** Baseline and follow-up correlation for the 7 low PiB subjects who showed increasing PiB signal over 2 years—three crossing the 1.5 threshold. **c** Baseline and follow-up correlation for 8 cases in the low PiB subgroup who showed no increase in PiB uptake over 2 years (*P* < 0.05; cluster FWE rate, *P* < 0.05)

Fifteen of our MCI cases were categorised as low/normal PiB at baseline and showed cortical surface areas where PiB uptake and levels of PK11195 uptake were positively correlated at baseline and again 2 years later. The cortical areas of positive correlation between PiB and PK11195 uptake became more extensive at the 2-year follow-up compared to the baseline findings, Fig. 2a. In this low-PiB MCI group, 7 of the 15 subjects showed increasing cortical PiB SUVR values at a 2-year follow-up, and three SUVRs now exceeding the 1.5 threshold for raised amyloid and so had formally converted to pAD [31]. When examining the positive correlation between PiB and PK11195 uptake in those 7 subjects with rising PiB uptake (Fig. 2b), we found a similar pattern of positive correlation as for the entire group of 15 low PiB subjects. In contrast, the 8 subjects who had stable low PiB SUVR values over 2 years showed no significant areas of positive correlation between PiB and PK11195 uptake (Fig. 2c). These findings implied the low-PiB MCI group consisted of two separate populations: a group of seven early prodromal Alzheimer cases missed by applying a conventional 1.5 SUVR PiB threshold for prodromal AD and a group of eight subjects who did not have AD.

### Correlations between cortical uptake in the high PiB MCI group, at baseline and 2-year follow-up

#### Correlations of cortical PiB and PK11195 uptake in the high PiB group

**Fig. 3:**
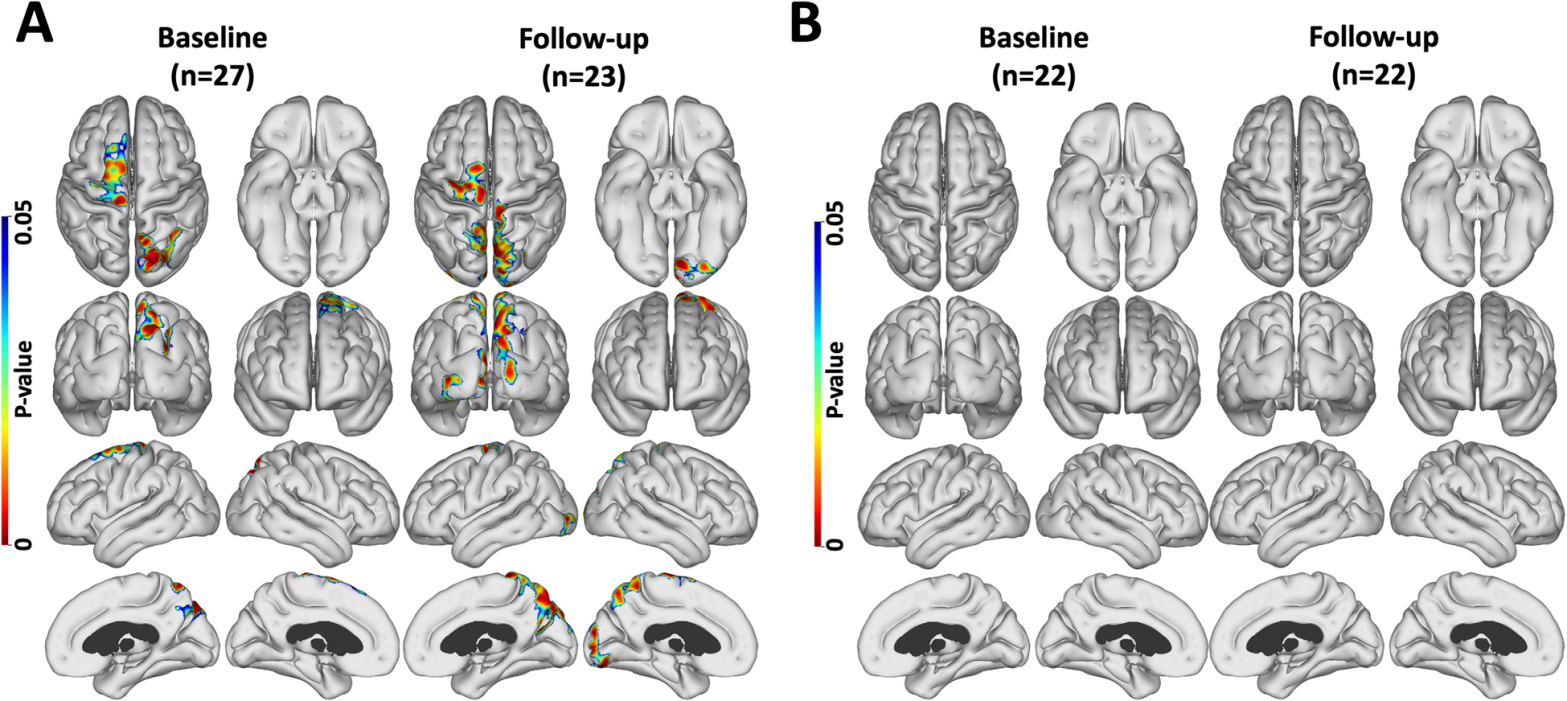
Amyloid and inflammation level correlation maps. Regions where levels of PiB and PK uptake were positively correlated **a** across high PiB MCI subject at baseline and after 2 years of follow-up. **b** Baseline and follow-up correlations after correction for tau influence across 22 subjects who had all three tracers. *P* < 0.05; cluster FWE rate, *P* < 0.05. Tau load influences the correlation between amyloid and inflammation levels

At baseline, scattered areas where cortical levels of PiB and PK11195 binding were positively correlated and were detected in frontal and parietal lobes across the high PiB group of 27 MCI (prodromal AD) subjects (Fig. 3a). Two years later, the returning 23 high PiB MCI subjects showed that the positively correlated PiB and PK11195 signals now had extended to involve the occipital cortex (Fig. 3a). No negative correlations between PiB and PK11195 levels were seen. However, the influence of tau load on the apparent correlation between cortical β-amyloid and inflammation levels still needed to be assessed. When we covaried out the effect of tau load (flortaucipir binding) on the association between PiB and PK11195 uptake levels in the 22 subjects who also had flortaucipir scans, the positive correlation between PiB and PK11195 uptake levels no longer remained statistically significant—see Fig. 3b. This implied that inflammation due to tau tangle formation could be influencing the apparent correlation between PiB and PK11195 levels in our high-PiB MCI cohort—see below.

#### Correlations between flortaucipir and PK11195 in the high PiB MCI group

**Fig. 4:**
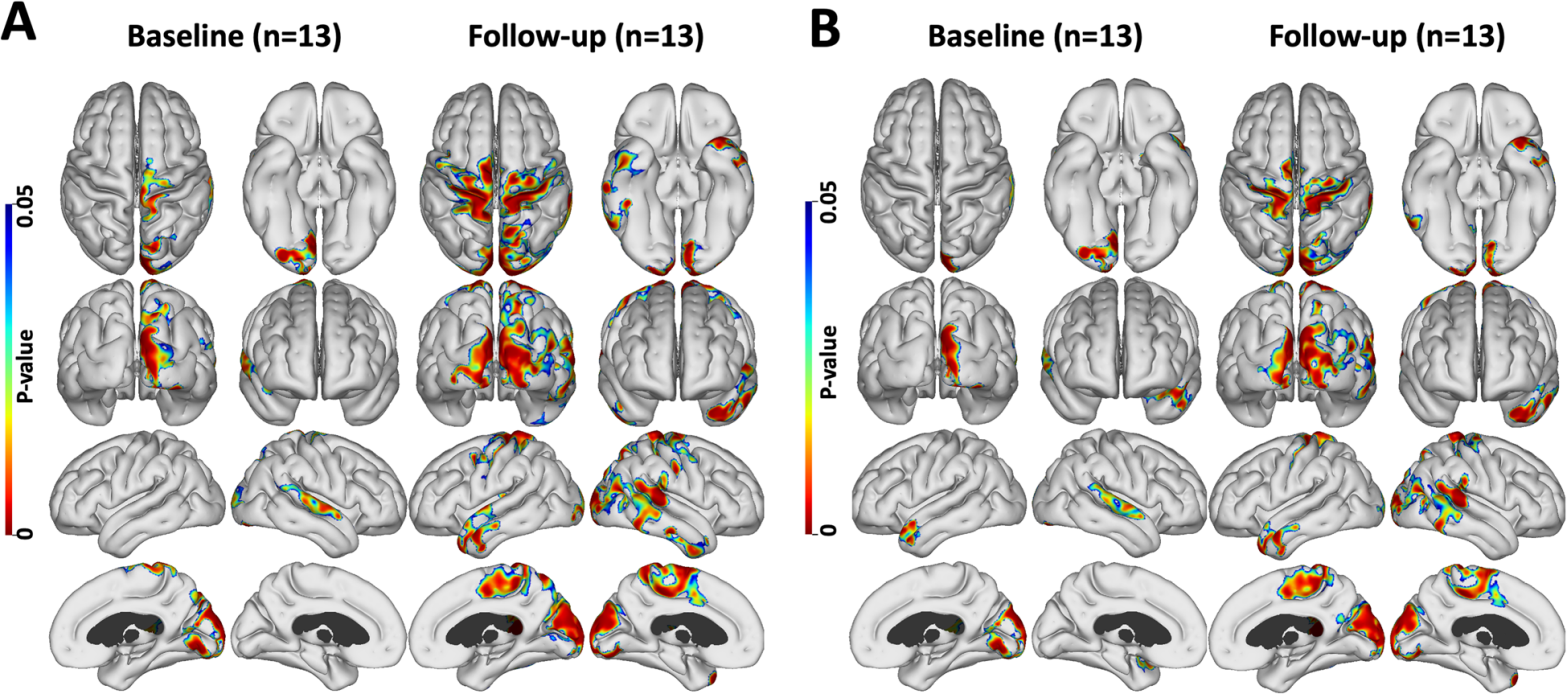
Tau and inflammation correlation maps in 13 high PiB MCIs. Positive correlation between flortaucipir and PK uptake. **a** Baseline and follow-up correlation in 13 high PiB MCI subjects. **b** baseline and follow-up correlation after correction for amyloid influence (*P* < 0.05; cluster FWE rate, *P* < 0.05). Amyloid load did not influence the correlation between tau and inflammation levels

Directly interrogating the association between flortaucipir and PK11195 levels, we found a positive correlation between levels of flortaucipir and PK11195 uptake in areas of cortex at both baseline and at a 2-year follow-up in the 13 high PiB MCI subjects who had all three PET tracers—see Fig. 4a. Flortaucipir and PK11195 levels were positively correlated in these cortical areas before and after covarying out the variance in PK11195 uptake associated with Aβ deposition (PiB binding). While the locations of cortical areas showing significantly correlated levels of flortaucipir and PK11195 uptake remained unchanged after covarying out amyloid effects, the extent of the correlations were smaller (Fig. 4b). This finding confirms that in high-PiB MCI, it is tau tangles that have the major influence on levels of inflammation.

#### The effect of PK11195 on the correlation between PiB and flortaucipir at baseline and follow-up

**Fig. 5:**
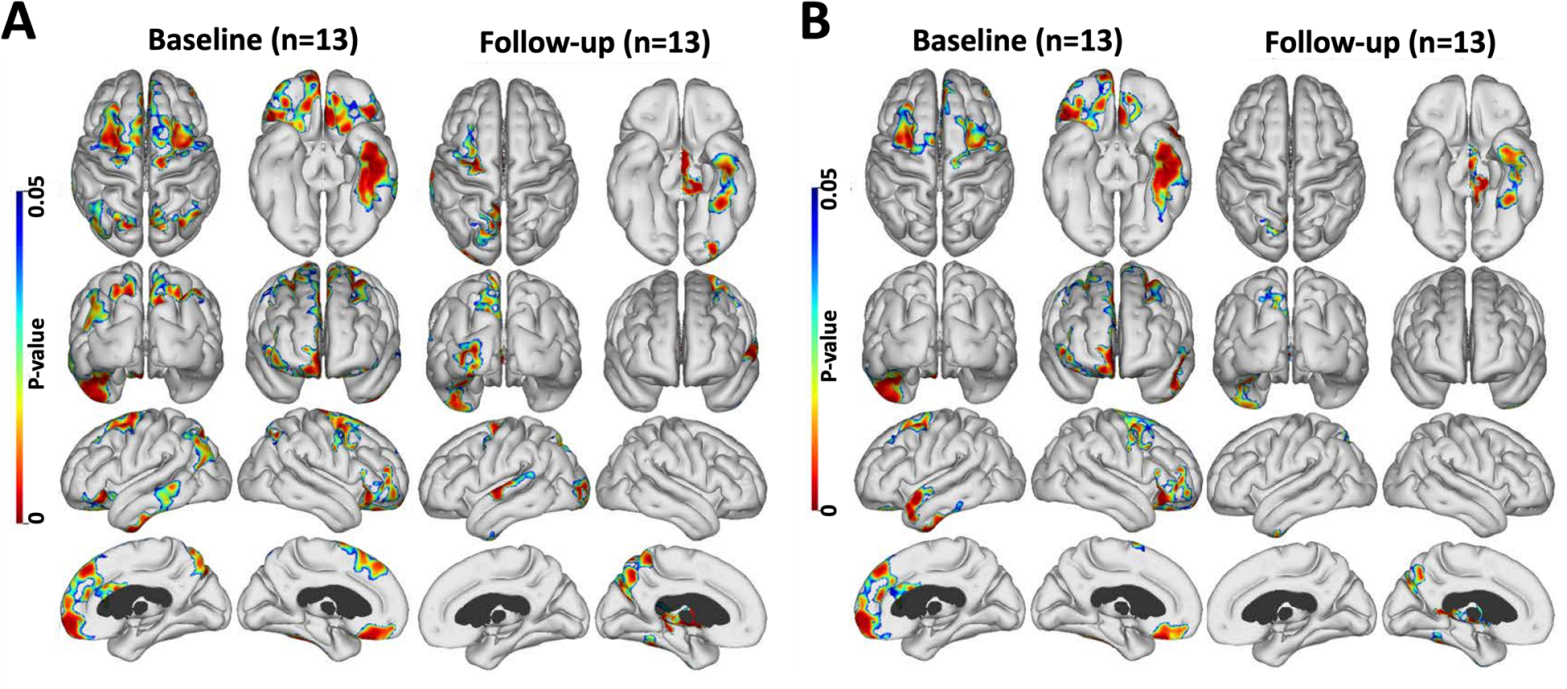
Tau and amyloid correlation maps in 13 high PiB subjects. **a** Positive correlation between PiB and flortaucipir levels in 13 high PiB subjects at baseline and after 2 years of follow-up. **b** The above correlation after correction for inflammation (*P* < 0.05; cluster FWE rate, *P* < 0.05). Inflammation did not influence the correlation between tau and amyloid levels

When interrogating the association between PiB and flortaucipir levels across 13 high PiB subjects, we found cortical areas of positive correlation between PiB and flortaucipir binding both at baseline and more so after 2 years of follow-up (Fig. 5a). The areas of correlation remained similar after correcting for any effect of inflammation (Fig. 5b). This suggests that inflammation does not act as the link between levels of amyloid and tau deposition.

## Discussion

Our longitudinal PET study was designed to investigate in vivo the spatial and temporal relationships between levels of neuroinflammation and the load of cortical Aβ and tau tangles at baseline and 2-year time points in MCI subjects. In this report, we have used a vector-based approach (FACE) to localise significant inter-correlations between the different imaging markers of Alzheimer’s pathology on the cortical surface. Our longitudinal data show that (#1) levels of cortical inflammation declined in temporal and frontal areas over 2 years in pAD cases (MCI cases with high PiB uptake at baseline) although their Aβ and tau loads were still rising (Fig. 1). Cross-sectional findings from other groups have also suggested a decline in inflammation levels as pAD progresses to clinical AD, the earliest pAD cases showing the highest inflammation levels [13, 32].

In our baseline low PiB MCI group, 7 cases showed a rise in Aβ load over 2 years (three into the Alzheimer range) and had levels of cortical inflammation that correlated with PiB uptake. This suggests that these low PiB MCI cases may initially have had early Thal phase 1 or 2 levels of Aβ present below our 1.5 SUVR threshold of abnormality and progressed over the 2 years associated with increased levels of inflammation—see Fig. 3 [31, 33, 34]. The 8 low PiB MCI cases which remained stable over 2 years showed no association between levels of PiB and PK11195 uptake and are unlikely to have Alzheimer’s disease.

Using voxel-based biological parametric mapping (BPM), we previously reported clusters of significant correlation between Aβ load and inflammation in MCI at baseline [15] but found no significant association between tau load and inflammation levels [16]. In this present study, we used FACE, a more sensitive vector-based cortical surface mapping approach to localise cortical surface areas where MCI cases with high PiB at baseline, and MCI cases with low PiB which rose after 2 years and had significant positive correlations between their Aβ load and inflammation levels—Figs. 2 and 3 [24]. While we were able to localise cortical areas where Aβ load and inflammation levels were positively correlated, in the high-PiB MCI group when we covaried out the effect of tau deposition on inflammation, the correlation between Aβ and inflammation levels was no longer statistically significant (Fig. 3). This suggests that it was the concomitant effect of the tau present on inflammation levels that was responsible for the apparent correlation between Aβ and inflammation levels in prodromal AD with an amyloid load approaching Alzheimer levels. This would not be surprising as Aβ levels plateau in Alzheimer’s disease and are approaching a steady level in the high PiB MCI group making correlations with inflammation difficult to demonstrate, whereas tau levels still continue to rise in these subjects.

(#2) Our findings support the view that inflammation can be present in the earliest prodromal Alzheimer disease stages as our 7 baseline low PiB MCI cases who showed rising PiB uptake over 2 years had correlated levels of Aβ and inflammation—Fig. 2. It has been reported that cortical Aβ needs to be present before tau tangles form [9]. Those of our baseline low PiB MCI group who had flortaucipir PET showed no evidence of tau tangle deposition [16] so tau is unlikely to be a confounder that could explain the correlated amyloid load and inflammation levels in these low PiB MCI cases.

(#3) In Alzheimer brain slices, activated microglia are seen surrounding both Aβ plaques and dying neurones containing tau tangles [1]. Post-mortem studies have also shown a positive correlation between activated microglia and NFT densities [2]. Our current findings support an association of tau tangles with raised inflammation levels in later prodromal stages of the disease— that is high PiB MCI cases— confirmed by our finding of a positive correlation between tau tangle load and inflammation levels across cortical areas at the 2-year follow-up. This correlation between tau and inflammation levels survived removal of the variance due to Aβ load on inflammation levels with ANCOVA even after applying an FWE correction to the cortical signals (Fig. 4). Comparing the localizations of cortical areas where PiB and PK11195 uptake were correlated with those where flortaucipir and PK11195 uptake were correlated, there was considerable overlap in their location, and these areas of correlation increased in extent over 2 years as the disease progressed.

The extended amyloid cascade hypothesis suggests that amyloid deposition leads to tau tangle formation with inflammation being a possible intermediate mediator of this chain. We have interrogated with ANCOVA the influence of inflammation levels on the positive correlation seen between amyloid and tau levels in our high PiB MCI cases (Fig. 5). We found that inflammation levels had no influence on the significance of the association between amyloid and tau loads. This is against inflammation driving tau tangle formation after Aβ deposition has occurred. We also explored the effect of Aβ levels on the correlation between tau and inflammation levels in our high PiB MCI cases and found no influence. In contrast, tau had a significant influence on the association between Aβ and inflammation levels in high-PiB MCI. Overall, our findings suggest that inflammation is more likely to be a direct response to Aβ and tau deposition than a cause of either of these abnormal protein aggregations.

The finding of inflammation in early MCI cases has been suggested to play a protective role by removing Aβ fibrils. If so, stimulation of microglial activation could possibly be advocated as a protective strategy in early prodromal AD cases, perhaps even before clinical symptoms occur. Immunotherapy would be a reasonable approach for achieving this in early disease stages.

Later in prodromal and early Alzheimer’s disease, the activated microglia fail to clear Aβ plaques and decline in number. In established Alzheimer’s disease, cortical fibrillar Aβ load reaches a plateau, and at this time, tau tangle load starts to rise facilitated by the presence of Aβ plaques in some manner that remains unclear. There is then a second wave of inflammatory activity in MCI which we and others have shown correlates with tau tangle levels [13]. It is proposed that these microglia have a neurotoxic phenotype and a microglial suppressant to reduce cytokine levels could be a rational protective intervention.

## Limitations of this study

This is a single centre study with a relatively low number of cases compared to multicentre series such as ADNI. We used a first generation TSPO PET tracer, ^11^C-PK11195, to detect inflammation, which has a higher background signal than second generation ligands but has the advantage that its binding is not influenced by the TSPO polymorphisms expressed by subjects. Specific PK11195 uptake was modelled by extracting a tissue non-specific reference cluster of voxels representing the kinetics of normal cortical grey matter but this reference cluster can contain voxels with low levels of specific binding occasionally leading to cortical voxels with apparent negative binding. ^11^C-PK11195 PET is strictly a marker of translocator protein (TSPO) expression and not microglial density. Elevations in TSPO are a non-specific reaction to inflammation, and TSPO is also expressed by cells other than microglia, including activated astrocytes [35]. One, therefore, has to be cautious in interpreting TSPO rises as entirely due to inflammation. TSPO is also expressed by vascular endothelia and the choroid plexus. The SVCA modelling approach we used filters out the kinetic components of tracer uptake by endothelial and choroidal epithelium cells though it cannot separate activated astrocyte and microglial signals. Post-mortem studies [35, 36] suggest, however, that the major TSPO signal comes from microglia in Alzheimer’s disease, but we acknowledge that some of the signal may also reflect activation of astrocytes. It seems reasonable to consider TSPO signal as a marker of intrinsic neuroinflammation.

We used ^18^F-Flortaucipir PET as our marker of paired helical tau but this modality shows off-target binding to iron rich proteins and melanin primarily seen subcortically [37].

## Conclusions

In summary, we have investigated at baseline and at a 2-year follow-up the relationship between inflammation levels and the loads of Aβ and tau across a group of MCI cases, the majority of whom had prodromal Alzheimer’s disease. Using an improved vector-based approach for localising significant changes in tracer uptake at cortical surfaces, we have found that (a) levels of inflammation decline in prodromal AD cases. (b) In MCI cases with low/normal cortical PiB uptake who subsequently show increased amyloid deposition over 2 years, levels of inflammation correlate with their Aβ load. (c) Conversely, MCI cases which had a high Aβ load approaching Alzheimer levels at baseline on their PiB PET, overall levels of inflammation declined over 2 years. Additionally, there was an association between tau rather than amyloid load and levels of inflammation in these subjects. These findings are compatible with a two-peak hypothesis of inflammatory activity in Alzheimer’s disease, and the first peak being driven by amyloid aggregation and possibly having a protective role and the second being driven by tau tangle formation and being neurotoxic (a). This suggests that one cannot simply boost or suppress inflammation as a protective strategy in Alzheimer’s disease—a rational treatment approach to stimulating or suppressing inflammation that will depend on whether brain regions in each subject are in the earliest phase of AD where microglia may be protective or in a later phase of the disease where they may have a neurotoxic phenotype.

## Supplementary information

**Additional file 1: Figure S1.** FSlow chart of the study.

## Data Availability

The datasets used supporting the conclusions of this article are available from the corresponding author on reasonable request.

## Abbreviations

AD: Alzheimer’s disease
Aβ: Beta-amyloid
BP_ND_: Binding potential (non-displaceable)
CDR: Clinical dementia rating
FACE: Fast accurate cortex
Flortaucipir: ^18^F-Flortaucipir
FWER: Family wise error rate
GM: Grey matter
MBq: Megabecquerel
MCI: Mild cognitive impairment
MoCA: Montreal cognitive assessment
PET: Position emission tomography
PiB: ^11^C-Pittsburgh compound B
PK11195: ^11^C-(R)-PK11195
TSPO: Translocator protein
SOB: Sum of boxes
