# Supplementary material for: The relationships between neuroinflammation, beta-amyloid and tau deposition in Alzheimer’s disease: a longitudinal PET study
Source: J Neuroinflammation. 2020 May 6;17:151. doi: 10.1186/s12974-020-01820-6 (PMC7203856; doi:10.1186/s12974-020-01820-6)
Supplement: Supplementary file 1 — Additional file 1: Figure S1. FSlow chart of the study. [file 12974_2020_1820_MOESM1_ESM.docx]

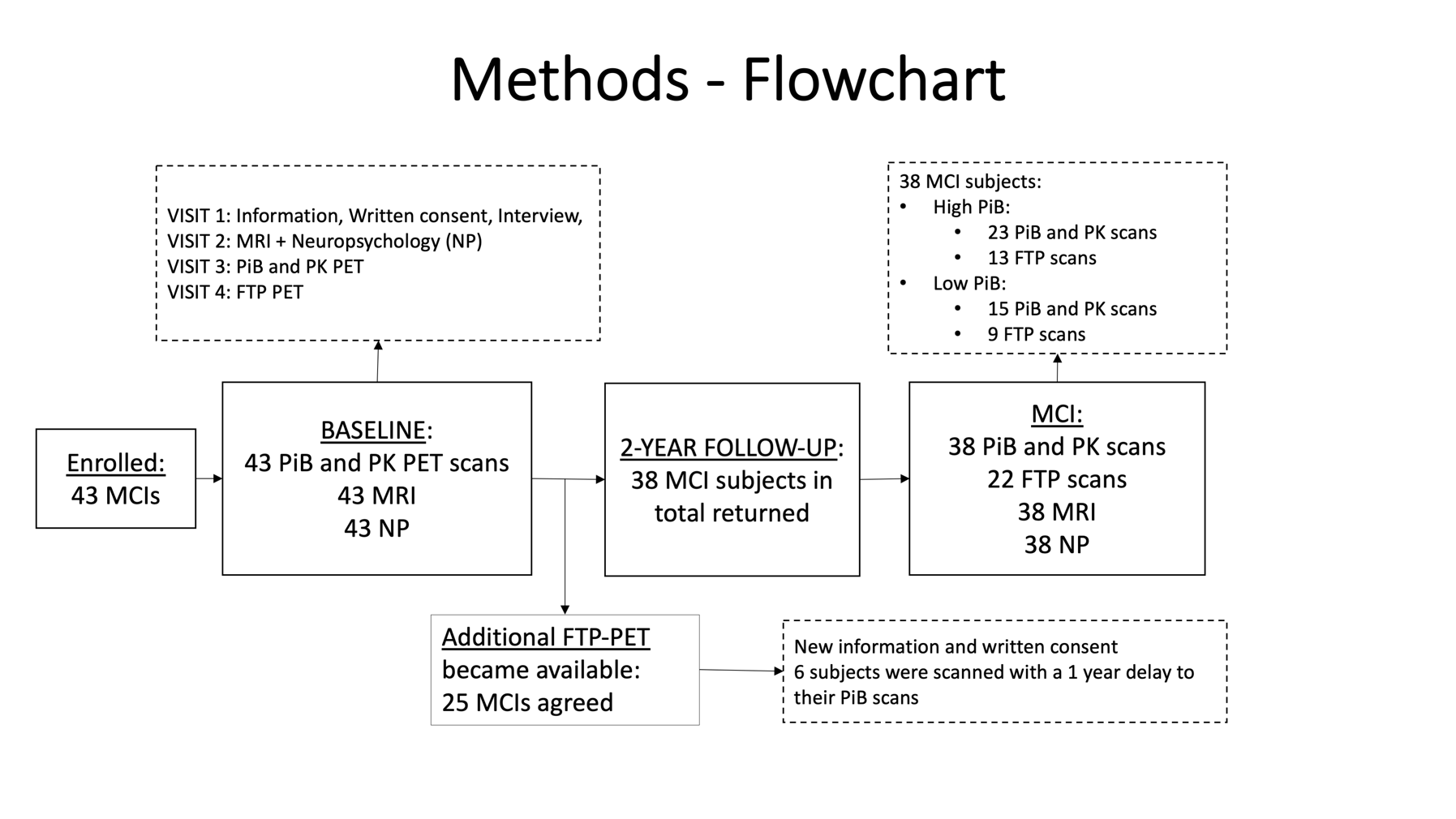


Additional file: Flowchart.

MCI: Mild Cognitive impairment; PiB: Pittsburgh Compound B; PK: PK11195; NP: Neuropsychology; FTP: Flortaucipir.
